# LncRNA MEG3 Restrains Hepatic Lipogenesis via the FOXO1 Signaling Pathway in HepG2 Cells

**DOI:** 10.1007/s12013-024-01278-w

**Published:** 2024-05-07

**Authors:** Xiangyu Meng, Mei Long, Nanxi Yue, Quan Li, Jia Chen, Hongye Zhao, Wei Deng

**Affiliations:** 1 The Central Laboratory, Beijing Jishuitan Hospital, Capital Medical University, Beijing, 100035 China; 2 Department of Cardiology, ZiBo Central Hospital, Zibo, Shandong 255000 China; 3 Department of Biochemistry and Molecular Biology, The Key Laboratory of Neural and Vascular Biology, Ministry of Education of China, Hebei Medical University, Shijiazhuang, Hebei 050017 PR China; 4 Department of Endocrinology, Beijing Jishuitan Hospital, Capital Medical University, Beijing, 100035 China

**Keywords:** NAFLD, LncRNA MEG3, FOXO1, Lipid metabolism

## Abstract

Nonalcoholic fatty liver disease (NAFLD) become a main public health concern, and is characterized by lipid accumulation in the hepatocytes. We found that overexpression of lncRNA MEG3 significantly reduced the expression of FOXO1, ACC1, and FAS, and subsequently decreased the lipid accumulation in HepG2 cells. Moreover, inhibition of lncRNA MEG3 could increase the lipid accumulation and the mRNA and protein levels of FOXO1, ACC1, and FAS. Further study showed that lncRNA MEG3 regulates the lipogenesis process by inhibiting the entry of FOXO1 into the nucleus translocation. Our study demonstrated that lncRNA MEG3 regulates de novo lipogenesis by decreasing the expression and nucleus translocation of FOXO1 in HepG2 cells, suggesting that lncRNA MEG3 could be a promising therapeutic target in lipid metabolic disorders.

## Introduction

Nonalcoholic fatty liver disease (NAFLD), a critical public health concern in the 21st century, afflicts nearly 20% of individuals in developed nations. As a metabolic syndrome, NAFLD is characterized by the accumulation of lipids in hepatocytes, particularly triglycerides (TG) [1]. It has been proposed that various factors, including insulin resistance, hyperlipidemia, hypertriglyceridemia, and oxidative stress, contribute to chronic inflammation in hepatocytes. This inflammation, in turn, triggers the activation of lipid metabolism signaling pathways, leading to increased lipid synthesis and subsequent hepatic steatosis and NAFLD [2, 3]. However, the specific mechanism underlying the genesis and development of NAFLD remains elusive.

FOXO1 belongs to the forkhead family of transcription factors which is the main target of insulin signaling and regulates metabolic homeostasis. The phosphatidylinositol 3 kinase (PI3K)/Akt pathway phosphorylates FOXO1 and mobilizes it from the nuclei to the cytoplasm, leading to inactivation of FOXO1 [4, 5]. In liver, FOXO1 regulation of Chrebp O-glycosylation, leads to the hepatic glucose utilization with lipid synthesis [6]. However, FOXO1 nuclear retention enhances lipid uptake and lipolysis, and potentiates UCP1 expression [7].

Chronic inflammation plays a pivotal role in lipid metabolism pathogenesis, and tumor necrosis factor (TNF)-α, promoting de novo lipogenesis both in vitro and in vivo. Studies showed that TNF-α mediates lipogenic signaling and the biosynthesis of non-esterified fatty acids and triglycerides through several signal transduction pathways [8, 9]. TNF-α could increase serum triglycerides by inducing stimulation of hepatic lipid synthesis in an insulin independent manner and enhancing lipid droplets formation via promoting phosphorylation of c-Jun N-terminal kinase (JNK), thus activating fatty acid synthase (FAS) and SREBP-1 [10, 11], resulting in an increase in de novo synthesis of fatty acids.

The lncRNA MEG3, initially discovered in glioma, is known to inhibit cell proliferation by recruiting and activating p53 through specific secondary structures [12, 13]. Recent research has also reported its involvement in lipid metabolism. Through promoting the ubiquitination and degradation of the enhancer of Zeste homolog 2 (EZH2), overexpression of lncRNA MEG3 suppresses lipid accumulation induced by free fatty acids (FFA), ultimately upregulating the expression of Sirtuin 6 (SIRT6) in hepatocytes [14].

In this study, we attempt to clarify the expression and regulation of lncRNA MEG3, TNF-α, and lipogenesis genes in HepG2 cells and explore possible intervention mechanism, providing new evidence for the prevention and treatment of NAFLD.

## Materials and Methods

### Cell Culture and Nuclear Protein Extraction

HepG2 cells were grown in minimum Eagle’s medium (MEM, Gibco) supplemented with 10% fetal bovine serum (FBS, Gibco), 100 U/mL penicillin G sodium and 100 mg/mL streptomycin sulfate in a humidified atmosphere with 5% CO_2_ at 37 °C. For TNF-α treatment, HepG2 cells were exposed to 10 ng/ml TNF-α for 24 h as described previously [15]. The nuclear protein was obtaied using the nuclear cytoplasmic extraction kit (Thermo, NE-PER) according to the manufacturer’s protocol.

### Adenovirus Vector Construction

The adenovirus vector expressing lncRNA MEG3 (Ad-MEG3) and the corresponding control adenovirus vector (Ad-Con) were purchased from GeneChem (Shanghai). HepG2 cells were infected with adenovirus at multiplicity of infection (MOI) 100.

### siRNA Infection

Transfection Synthetic specific siRNA of lncRNA MEG3 (siMEG3) and FOXO1 (siFOXO1) were obtained from GenePharma (Shanghai). Cells were transfected at 100 nM with specific siRNA targeting FOXO1 or lncRNA MEG3 using Lipofectamine® 2000 Transfection Reagent (Life Technologies, NY, USA), according to the manufacturer’s instructions. Non-targeting siRNA oligomers were used as negative control (NC).

### RNA Isolation and Real-time PCR

Total RNA was extracted from cells using TRIzol reagent (Invitrogen, Carlsbad, California, USA), according to the manufacturer’s instructions. SYBR Green I (TaKaRa) was used for real-time PCR according to the manufacturer’s instructions with the ABI 7500 qPCR System (Life Technologies, CA). The relative expression level of mRNA was normalized to β-actin. Each reaction was performed in triplicate, and analysis was conducted using the 2^-ΔΔCT^ method. The primer sequences were listed in Table 1.

**Table 1:** Primers and protocols for qRT-PCR

Genes	Primer sequence
Human lncRNA MEG3	Fwd: 5′-TGCTGCCCATCTACACCT-3′
	Rev: 5′-CTTCATCCTTTGCCATCC-3′
Human FOXO1	Fwd: 5′-TCGTCATAATCTGTCCCTACACA-3′
	Rev: 5′-CGGCTTCGGCTCTTAGCAAA-3′
Human ACC1	Fwd: 5′-GCACATAAGGTCCAGCAT-3′
	Rev: 5′-CCCAAAGCGAGTAACAAA-3′
Human FAS	Fwd: 5′-GCCCGCTCTGGTTCATCT-3′
	Rev: 5′-CGGTTCACAGCCTCATCG-3′
Human SREBP1c	Fwd: 5′-TTGCCGACCCTGGTGAGT-3′
	Rev: 5′-AATGGCGTTGTGGGCTGT-3′
Human β-actin	Fwd: 5′-CATGTACGTTGCTATCCAGGC-3′
	Rev: 5′-CTCCTTAATGTCACGCACGAT-3′

*FOXO1* Forkhead Box O 1, *ACC1* Acetyl-CoA carboxylase 1, *FAS* Fatty acid synthase, *SREBP1c* Sterol regulatory element binding protein-1c.

### Western Blot Analysis

Nuclear and cytoplasmic protein fractions were isolated using Nuclear Extraction kit (Abcam, Cambridge, MA, USA), according to manufacturer’s protocols. Cell lysates (10–30 μg protein) were separated using 10% SDS‑PAGE and transferred to polyvinylidene fluoride membranes (Millipore Corporation, Billerica, MA, USA), blocked by 5% non‑fat dry milk for 1 h and incubated with primary antibodies at 4 °C overnight. Followed by probing with horseradish peroxidase‑conjugated anti‑immunoglobuin G (ZSGB-Bio, Beijing, China). GelDoc XR system (Bio-Rad, Hercules, CA) was adopted in the detection and data collection. Rabbit monoclonal anti‑FOXO1, Anti-FAS, and anti‑β-actin antibodies were purchased from Abcam (Abcam, Cambridge, MA, USA). Antibody against ACC1 was obtained from Cell Signaling Technology, Inc. (Beverly, MA, USA).

### Immunofluorescence Analysis

HepG2 cells seeded in 6-wells plates were fixed with 4% formaldehyde for 10 min and permeabilized with 0.5% Triton X-100 for 15 min, then incubated with goat serum for 30 min. Followed by incubation with anti-FOXO1 antibody (1:200 in PBS) overnight at 4 °C. Then, cells were washed 3 times and incubated with TRITC conjugated goat anti-rabbit (594 nm, 1:200) antibody for 1 h. Nuclei were stained using DAPI (1:1000 diluted in PBS) for 5 min and examined with a fluorescence microscope (Olympus, Tokyo, Japan).

### Oil Red O Staining

HepG2 cells were fixed with 4% formaldehyde for 10 min, then incubated with prewarmed Oil Red O (Solarbio, Beijing, China) for 30 min and washed with 60% propanediol for about 10 s. The lipid droplets were observed under a microscope (Olympus, Tokyo, Japan).

### Intracellular TG Measurement

The intracellular triglyceride was measured as described previously [16]. Cells were washed three times with cold PBS and extracted with CHCl3/MeOH (2:1), and then added 0.05% H_2_SO_4_ and centrifuged. The precipitation was transferred to 1% Triton X‑100 (1:1, v/v), dried, and dissolved in deionized water, and the content of intracellular triglycerides was measured using a triglyceride enzymatic assay kit (SSYF Medical Diagnostic Products Co., Ltd., Shanghai, China).

### Statistics

All data are presented as the means ± SD of the indicated number of measurements. Differences were analyzed using the Student’s *t*‑test. *P* < 0.05 was considered a statistically significant difference.

## Results

### LncRNA, MEG3, and Lipogenesis Genes were Upregulated in TNF-α Treated HepG2 Cells

**Fig. 1:**
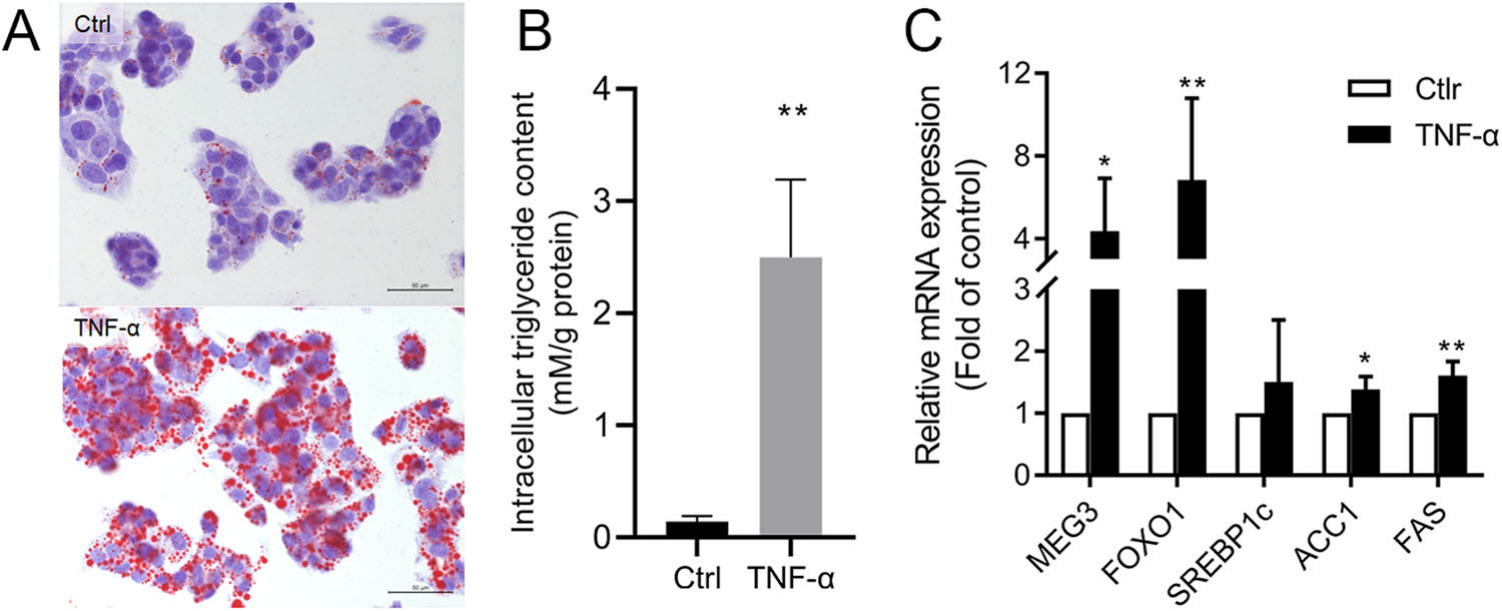
TNF-α induced the expression of lncRNA MEG3 and lipogenesis genes in HepG2 cells. **A** Oil Red O staining of HepG2 cells treated with 10 ng/ml TNF-α for 24 h (Scale bar = 50 μm). **B** The RNA expression of lncRNA MEG3 and the mRNA level of lipogenesis genes. *P < 0.05, **P < 0.01 vs blank. Data are mean ± SD, *N* = 3 independent experiments

In the previous study, we confirmed TNF-α could induce hepatic insulin resistance both in vitro and in vivo [15]. To further research on metabolic diseases, we still use HepG2 cells and treat them with TNF-α (10 ng/ml) for 24 h, and intracellular lipid accumulation increased significantly treated with TNF-α (Fig. 1A, B), Meanwhile, the expression of lipogenesis genes such as Acetyl-CoA carboxylase 1 (ACC1), FAS, Forkhead box protein 1 (FOXO1), as well as lncRNA MEG3 significantly increased (Fig. 1C).

### Overexpression of lncRNA MEG3 Restrained Lipogenesis Gene

**Fig. 2:**
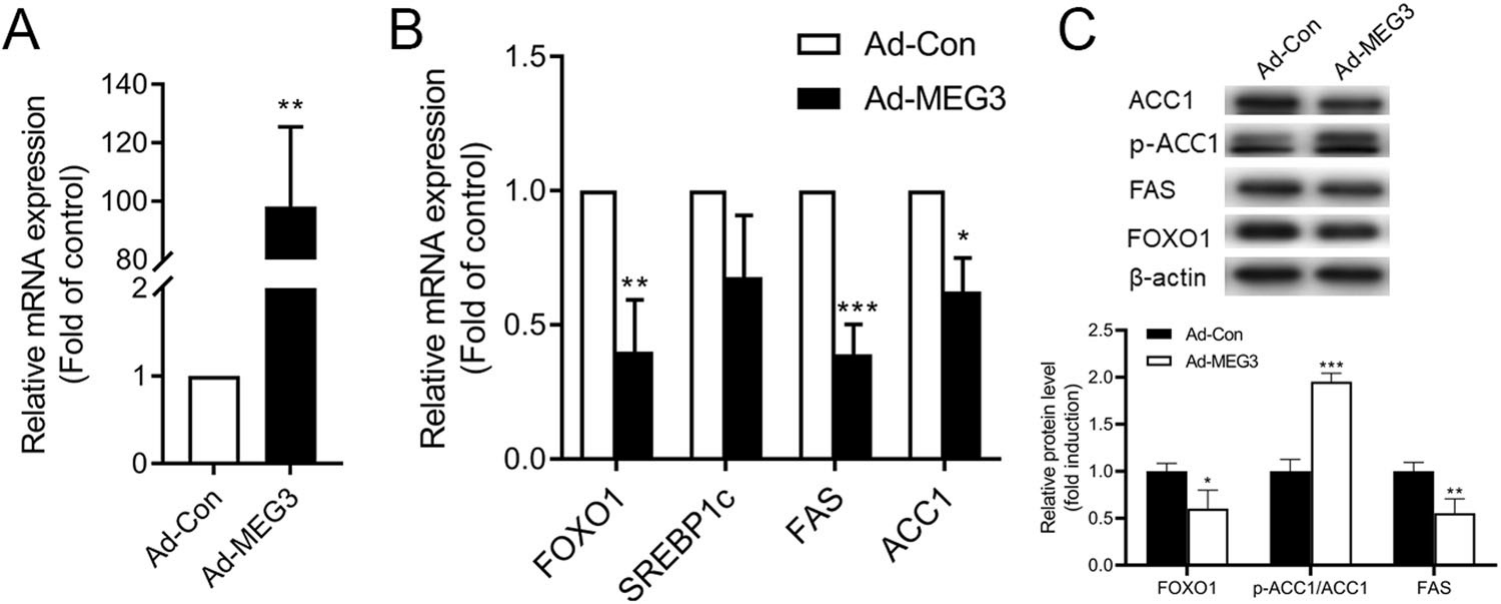
LncRNA MEG3 inhibited the expression of lipogenesis genes. **A** qPCR assay for intracellular expression of lncRNA MEG3 after adenovirus vector (Ad-MEG3) transfected 48 h. **P < 0.01 vs Ad-Con. Data are mean ± SD. *N* = 6 independent experiments. **B** qPCR assay for FOXO1 and lipogenesis genes. *P < 0.05 vs Ad-Con, **P < 0.01 vs Ad-Con ***P < 0.001 vs Ad-Con. Data are mean ± SD. *N* = 6 independent experiments. **C** Western blot assay for the expression of FOXO1, ACC1, and FAS in HepG2 cells. *P < 0.05 vs Ad-Con, **P < 0.01 vs Ad-Con, ***P < 0.001 vs Ad-Con. Data are mean ± SD, *N* = 3 independent experiments

To determine whether lncRNA MEG3 is promoted in lipogenesis, we upregulated the intracellular lncRNA MEG3 by delivering a specific adenovirus vector (Ad-MEG3) (Fig. 2A). The overexpression of lncRNA MEG3 repressed the mRNA expression and the protein level of FOXO1, along with the de novo lipogenesis markers ACC1 and FAS. It also gives rise to upregulated phosphorylation of ACC1 (Fig. 2B, C). These results indicated that the overexpression of lncRNA MEG3 leads to inhibition of the de novo lipogenesis signaling pathway.

### Inhibited lncRNA MEG3 Enhanced the Lipogenesis in HepG2 Cells Treated with TNF-α

**Fig. 3:**
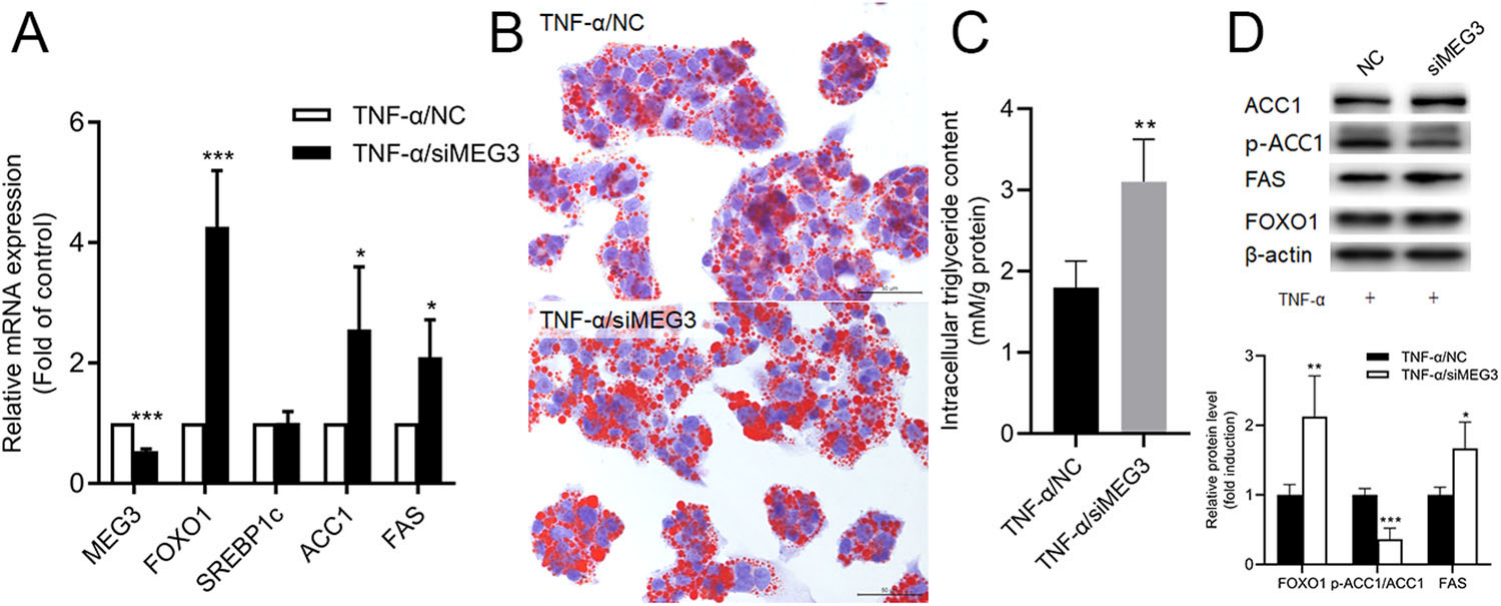
inhibition of lncRNA MEG3 could induce the expression of FOXO1 and lipogenesis genes in HepG2 cells. **A** qPCR assay for expression of lncRNA MEG3, FOXO1, SREBP1c, ACC1, and FAS in HepG2 cells. *P < 0.05 vs NC, ***P < 0.001 vs NC. Data are mean ± SD, *N* = 3 independent experiments. **B** Oil Red O staining of HepG2 cells transfected with siRNA targeted lncRNA MEG3 for 48 h (Scale bar = 50 μm). **C** Intracellular TG content assay. Data are mean ± SD, *N* = 3 independent experiments. **P < 0.01 vs NC. **D** Western blot assay for the expression of FOXO1, ACC1, and FAS in HepG2 cells treated with TNF-α. *P < 0.05 vs NC, **P < 0.01 vs NC, ***P < 0.001 vs NC. Data are mean ± SD, *N* = 3 independent experiments

Next, by silencing specific siRNA, we knocked down the expression of lncRNA MEG3 in HepG2 cells treated with TNF-α (Fig. 3A). The results showed that intracellular TG increased after the knockdown of lncRNA MEG3 (Fig. 3B, C). Furthermore, the mRNA and protein levels were upregulated by FOXO1, ACC1, FAS. As well as the suppression of the phosphorylation of ACC1. (Fig. 3A, D). These results suggested that inhibiting the lncRNA MEG3 could further promote the de novo lipogenesis in HepG2 cells under the stimulation of TNF-α.

### LncRNA MEG3 Regulated the Expression of Lipogenesis Via FOXO1 Signaling Pathway in HepG2 Cells

**Fig. 4:**
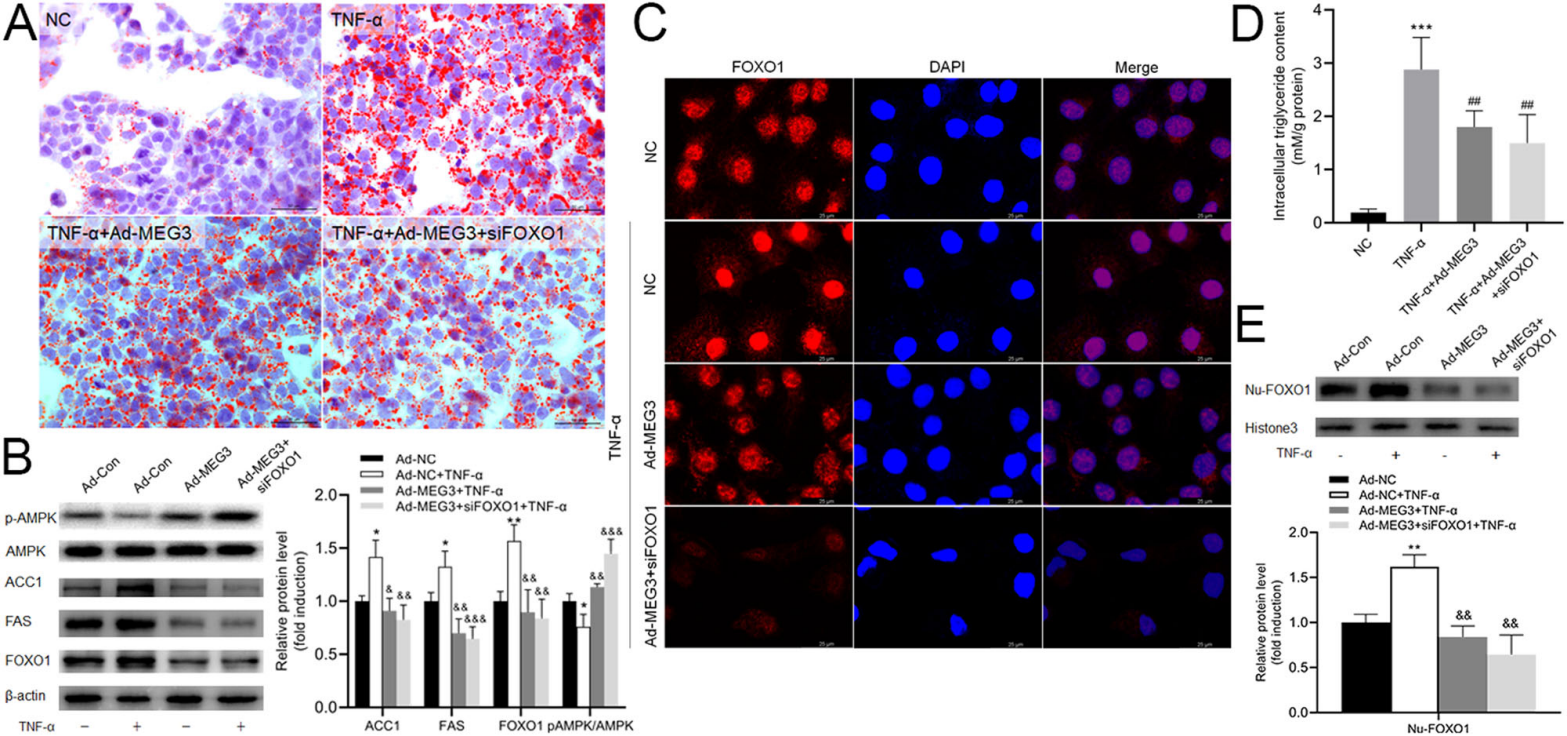
LncRNA MEG3 regulated the expression of lipogenesis via FOXO1 signaling pathway in HepG2 cells. **A** Oil Red O staining of HepG2 cells transfected with adenovirus vector and/or siRNA targeted lncRNA MEG3 for 48 h (Scale bar=50 μm). **B** Western blot assay for FOXO1, ACC1, and FAS transfected lncRNA MEG3 adenovirus vector and/or siRNA targeted FOXO1 for 48 h in HepG2 cells. **P < 0.01 vs Ad-NC, **P < 0.01 vs Ad-NC, ^&^*P* < 0.05 vs Ad-MEG3, ^&&^*P* < 0.01 vs TNF-α, ^&&&^*P* < 0.001 vs TNF-α. Data are mean ± SD, *N* = 3 independent experiments. **C** Representative immunofluorescence staining with antibodies for FOXO1 in HepG2 cells transfected with adenovirus vector and/or siRNA targeted lncRNA MEG3 for 48 h (Scale bar = 25 μm). **D** Intracellular TG content assay. Data are mean ± SD, *N* = 3 independent experiments. ***P < 0.001 vs NC, ^##^*P* < 0.01 vs TNF-α. **E** Western blot assay for the expression of FOXO1 in the nucleus of HepG2 cells transfected with lncRNA MEG3 adenovirus vector and/or siRNA targeted FOXO1 for 48 h. *P < 0.05 vs Ad-NC, ^&&^*P* < 0.01 vs Ad-MEG3. Data are mean ± SD, N = 3 independent experiments

In addition, to further explore whether lncRNA MEG3 regulates lipogenesis via FOXO1 signaling pathway, we deployed siRNA targeted FOXO1 and Ad-MEG3 in the following section of this study. Results displayed that overexpression of lncRNA MEG3 could reverse the cellular TG accumulation induced by TNF-α (Fig. 4A, D). Combining with inhibition of FOXO1 could not further reduce the intracellular TG content. In addition, the protein upregulation of ACC1, FAS, phosphorylated AMPK and FOXO1 by TNF-α could be reversed by the overexpression of lncRNA MEG3. There is no significant difference when combine with the inhibition of FOXO1 is compared to overexpression of lncRNA MEG3 alone (Fig. 4B). We also found that TNF-α could significantly increase the nuclear fluorescence staining of FOXO1, and increased lncRNA MEG3 or inhibition of FOXO1 could significantly reduce the nuclear FOXO1 fluorescence (Fig. 4C). Moreover, the increased intracellular lipid accumulation treated with TNF-α could be reversed by lncRNA MEG3 and lncRNA MEG3 combined with siFOXO1, respectively (Fig. 4D). The increase of lncRNA MEG3 also could reverse the nuclear FOXO1 induced by TNF-α (Fig. 4E). These results suggested that lncRNA MEG3 restrained de novo lipogenesis by inhibiting the expression and nucleus translocation of FOXO1.

## Discussion

LncRNA is a kind of non-coding RNA with transcripts more than 200 bp in length, most of which are unclear in function. It has been indicated that lncRNA becomes involved in chromosome recombination, gene modification, epigenetic processes, as well as synthesis and regulation of protein [17–21]. However, there are few studies reporting the role of lncRNA in triglyceride accumulation in hepatocytes. Therefore, the study of the function and mechanism of lncRNA in lipid metabolism could not only provide insights into the regulation of the human genome, but also facilitate the elucidation of the pathogenesis of NAFLD. Studies showed CRE-binding protein (CREB) binds directly to cAMP response element (CRE) site and stimulates the promoter activity of lncRNA MEG3 [22]. Hence, lncRNA MEG3, as the downstream target gene of cAMP, could be intimately associated with cellular energy metabolism. Our study suggested that an elevated level of lncRNA MEG3 could be observed under the stimulation of inflammation and fatty acids in HepG2, while suppression of lncRNA MEG3 could inhibit the expression of genes associated with lipid synthesis, and overexpression of lncRNA MEG3 could increase the levels of synthesis factors of fatty acids accordingly, such as ACC1 and FAS, which revealed that lncRNA MEG3 might play a role in the lipid metabolism pathways.

TNF-α is an important lipid metabolism regulator and plays avital role in lipogenesis. Many signaling pathways might be involved in TNFα-mediated lipid metabolism. Early studies have demonstrated that TNF-α could induce the rapid stimulation of hepatic FFA de novo synthesis in normal rats. In 3T3-L1 adipocytes, TNF-α promotes NF-κB pathway resulting in IR and increase of plasma FFA in rat liver [23, 24]. In addition, TNF-α could also regulate a wide range of lipogenesis enzyme activities and pathways such as ERK/JNK to cAMP, which led to the de novo lipogenesis and the accumulation of lipids [8]. Marathon running exercise induced an increase in plasma FFA, IL-6, and TNF-α, which afterwards induced plasma ANGPTL4 release, FFA-induced lipotoxicity and oxidative stress [25]. Our results showed that under the stimulation of 10 ng/ml of TNF-α, the intracellular TG content of HepG2 cells significantly increased, accompanied by upregulation of lncRNA MEG3 expression and increased mRNA and protein expression of lipid metabolism genes such as FAS, FOXO1, and ACC1. To clarify whether the increase in lncRNA MEG3 is due to the inhibitory factor of TNF-α which caused abnormal lipid metabolism or promoted changes in lipid metabolism pathways, we inhibited the expression of lncRNA MEG3 on the basis of TNF-α stimulation. The results showed that after inhibiting lncRNA MEG3, the TG content of HepG2 cells further increased, and the mRNA and protein expression of ACC1, FAS, FOXO1 significantly increased. It indicated that lncRNA MEG3 can be considered as an inhibitory factor of lipid metabolism.

FOXO1 is widely distributed in adult tissues and organs and becomes involved in energy metabolism and oxidative stress. An elevated expression of lncRNA MEG3 could be detected in the livers of ob/ob mice and diet-induced obese mice [26]. Overexpression of lncRNA MEG3 in primary liver cells could increase the mRNA levels of FOXO1, phosphoenolpyruvate carboxykinase (PEPCK) and G6Pase, enhance gluconeogenesis, and suppress the glycogen synthesis induced by insulin, whereas the suppression of lncRNA MEG3 could reverse this process. It has been suggested that FOXO1 can induce the expression of microsomal triglyceride transfer protein (MTP) by binding to the promoter of MTP. MTP assembles very low density lipoprotein (VLDL) together with apoB to become involved in the secretion of TG in hepatocytes. Suppression of FOXO1 could down-regulate the expression MTP and VLDL [27]. Local high levels of TNF-α could activate FOXO1 in the process of endochondral ossification in patients with fractures associated with diabetes, leading to an increased mRNA expression level of apoptosis gene and chondrocyte apoptosis [28]. It implied that FOXO1 could become involved in hepatic glycolipid metabolism by affecting inflammatory reaction in many ways. Mammalian ACC1 catalyzes the carboxylation of acetyl-CoA to form malonyl-CoA, an intermediate in the de novo synthesis of fatty acids [16]. Our study also suggested that TNF-α could result in an enhanced expression of lncRNA MEG3, an increase of FOXO1 translocation to the nucleus, an elevated level of ACC1, and increased lipid deposition in HepG2, while the overexpression of lncRNA MEG3 could reverse this process in part. It could be hypothesized that TNF-α can induce FOXO1 translocation into the nucleus to serve as a transcription factor, and then upregulate the expression of lipid synthesis factor ACC1 to stimulate intracellular lipid synthesis. LncRNA MEG3 is a factor that can inhibit lipid synthesis, but its elevation under the stimulation of TNF-α is not sufficient to inhibit the lipid synthesis process. Only additionally increase lncRNA MEG3 can reverse the lipid synthesis process caused by TNF-α.

There are several limitations in this study, we did not investigate the molecular mechanisms of lncRNA MEG3 and FOXO1. The intracellular glycometabolism was not explored either. The present study demonstrated that lncRNA MEG3 could become involved in lipid synthesis by regulating the expression and translocation to the nucleus of FOXO1, providing new insights into the molecular mechanism and control of NAFLD.

## Data Availability

No datasets were generated or analysed during the current study.
